# Pyrotinib in combination with letrozole for hormone receptor-positive, human epidermal growth factor receptor 2-positive metastatic breast cancer (PLEHERM): a multicenter, single-arm, phase II trial

**DOI:** 10.1186/s12916-023-02943-2

**Published:** 2023-06-26

**Authors:** Zhe-Yu Hu, Min Yan, Huihua Xiong, Li Ran, Jincai Zhong, Ting Luo, Tao Sun, Ning Xie, Liping Liu, Xiaohong Yang, Huawu Xiao, Jing Li, Binliang Liu, Quchang Ouyang

**Affiliations:** 1 Medical Department of Breast Cancer, Hunan Cancer Hospital, No. 283, Tongzipo Road, Changsha, 410013 China; 2 Medical Department of Breast Cancer, the Affiliated Cancer Hospital of Xiangya School of Medicine, Central South University, Changsha, China; 3 Department of Breast Cancer, Henan Cancer Hospital, Zhengzhou, China; 4 Department of Oncology, Tongji Hospital of Tongji Medical College of Huazhong University of Science and Technology, Wuhan, China; 5 Department of Oncology, The Affiliated Hospital of Guizhou Medical University, Guiyang, China; 6 Department of Medical Oncology, The First Affiliated Hospital of Guangxi Medical University, Nanning, China; 7 Department of Breast Surgery, West China Hospital, Sichuan University, Chengdu, China; 8 Cancer Hospital of Dalian University of Technology, Liaoning Cancer Hospital, Shenyang, China

**Keywords:** Hormone receptor-positive, HER2-positive, Metastatic breast cancer, Pyrotinib, Letrozole

## Abstract

**Background:**

Human epidermal growth factor receptor 2 (HER2) targeted therapy combined with endocrine therapy has been recommended as an alternative treatment strategy for patients with hormone receptor (HR)-positive, HER2-positive metastatic breast cancer (MBC). This study aimed to evaluate the role of pyrotinib, an oral pan-HER irreversible tyrosine kinase inhibitor, in combination with letrozole for patients with HR-positive, HER2-positive MBC.

**Methods:**

In this multi-center, phase II trial, HR-positive and HER2-positive MBC patients who were not previously treated for metastasis disease were enrolled. Patients received daily oral pyrotinib 400 mg and letrozole 2.5 mg until disease progression, unacceptable toxicity, or withdrawal of consent. The primary endpoint was the clinical benefit rate (CBR) assessed by an investigator according to the Response Evaluation Criteria in Solid Tumors version 1.1.

**Results:**

From November 2019 to December 2021, 53 patients were enrolled and received pyrotinib plus letrozole. As of August 2022, the median follow-up duration was 11.6 months (95% confidence interval [CI], 8.7–14.0 months). The CBR was 71.7% (95% CI, 57.7–83.2%), and the objective response rate was 64.2% (95% CI, 49.8–76.9%). The median progression-free survival was 13.7 months (95% CI, 10.7–18.7 months). The most common treatment-related adverse event of grade 3 or higher was diarrhea (18.9%). No treatment-related deaths were reported, and one patient experienced treatment discontinuation due to adverse event.

**Conclusions:**

Our preliminary results suggested that pyrotinib plus letrozole is feasible for the first-line treatment of patients with HR-positive and HER2-positive MBC, with manageable toxicities.

**Trial registration:**

ClinicalTrials.gov, NCT04407988.

## Introduction

A common malignancy affecting women worldwide, breast cancer poses a serious threat to their life and health [1]. Breast cancers with human epidermal growth factor receptor 2 (HER2) gene amplification or overexpression and hormone receptor (HR)-positive account for approximately 10% of all cases [1, 2]. Currently, HER2-targeted therapy combined with chemotherapy is recommended as the first-line treatment for metastatic breast cancer (MBC) with HER2 positivity, regardless of the HR status [3]. The combination of trastuzumab, pertuzumab, and docetaxel yielded a median progression-free survival (PFS) of 18.7 months, and a median overall survival (OS) of 57.1 months in the first-line setting in the CLEOPATRA study, which has been recommended as the preferred regimens for untreated HER2-positive MBC [4–6]. However, previous studies have shown that the use of chemotherapy is associated with a higher incidence of toxicities than endocrine therapy in HR-positive patients [7, 8].

There is widespread crosstalk between the HER2 and estrogen receptor (ER) signaling pathways, which contributes to resistance to both HER2-targeted therapy and endocrine therapy [9–11]. To overcome resistance, HER2-targeted agents have been investigated in combination with endocrine therapy, and the combination has yielded synergistic effects in patients with HER2-positive and HR-positive MBC patients. The phase III TAnDEM study suggested that trastuzumab plus anastrozole was feasible for HER2-positive and HR-positive MBC patients [12]. As noted in PERTAIN study, the combination of trastuzumab, pertuzumab, and an aromatase inhibitor (AI) improved the prognosis of HR-positive and HER2-positive MBC patients in the first-line setting, with a median PFS of 18.89 months [13]. Besides the use of monoclonal antibodies, small-molecular tyrosine kinase inhibitors (TKIs) also have an essential role in treating HER2-positive breast cancer. The convenience of oral administration of TKI agents promotes better patient compliance [14]. In a phase III randomized controlled trial, the combination of letrozole and lapatinib was investigated in patients with HER2-positive and HR-positive MBC in the first-line setting [15].

Pyrotinib is an irreversible small-molecular tyrosine kinase inhibitor (TKI), which targeted HER1, HER2, and HER4 [16]. Trials have shown that it is superior to lapatinib when combined with capecitabine in previously treated MBC with HER2-positive disease [17, 18]. However, the role of pyrotinib in combination of endocrine therapy has not been evaluated yet. Therefore, this study aimed to evaluate the feasibility of pyrotinib plus letrozole as a first-line treatment regimen to treat patients with HR-positive, HER2-positive MBC. Our results may provide preliminary evidence for future clinical trials.

## Methods

### Study design and patients

In this multicenter, single-arm, open-label phase II trial, patients with HER2-positive and HR-positive MBC who had not received treatment for metastasis disease from seven centers in China were included. The inclusion criteria were (1) aged 18 ~ 70 years; (2) histologically or cytologically confirmed MBC; (3) ER-positive confirmed by immunohistochemistry (IHC), with ≥ 10% positive cells (local laboratory assessment); (4) HER2-positive, defined as IHC 3 + or IHC 2 + with fluorescence in situ hybridization (FISH) positivity; (5) prior (neo)adjuvant trastuzumab, pertuzumab or chemotherapy were eligible; if indicated, the disease free interval (DFI) must be greater than 12 months from completion of (neo)adjuvant trastuzumab and pertuzumab; (6) patients either pre-, peri-, or post-menopausal were eligible, and ovarian function suppression (OFS) should be combined in case of pre- or peri-menopausal; (7) had at least one measurable metastatic disease according to the Response Evaluation Criteria In Solid Tumors, version 1.1 (RECIST v1.1) [19]; and (8) Eastern Cooperative Oncology Group (ECOG) performance status score of 0–1. The exclusion criteria included (1) with visceral crisis; (2) with central nervous system (CNS) metastases; (3) with factors that affect oral drug use and absorption; (4) patients who had received radiotherapy, endocrine therapy, chemotherapy, surgery (excluding local puncture) or targeted therapy in the advanced setting; (5) with other malignancies within 5 years; and (6) women who were pregnant or breastfeeding.

All enrolled patients provided the informed consent forms. The study was approved by the ethics committee of Hunan Cancer Hospital, and conducted in accordance with the Declaration of Helsinki, guidelines of the International Conference for Harmonization and Good Clinical Practice, as well as local ethical and legal requirements. This trial was registered at ClinicalTrials.gov (NCT04407988).

### Procedures

In eligible patients, oral pyrotinib 400 mg and letrozole 2.5 mg were administered daily until disease progression, unacceptable toxicity, or withdrawal of consent occurred. Prophylactic loperamide was not employed for preventing diarrhea. As soon as patients experienced grade 1 diarrhea, loperamide was administered at a dosage of 4 mg, followed by additional doses of 2 mg for every subsequent instance of loose stools, up to a maximum daily dose of 16 mg.

Enhanced computed tomography or magnetic resonance imaging was performed every 8 weeks to assess tumor response until disease progression or death in accordance with RECIST v1.1. A confirmation should be made in the next assessment. Adverse events (AEs) reported during the study were graded using the National Cancer Institute Common Terminology Criteria for Adverse Events (NCI-CTCAE) version 4.03.

### Outcomes

The primary endpoint was clinical benefit rate (CBR) according to the RECIST v1.1, which is defined as the proportion of patients who achieved confirmed complete response (CR), partial response (PR), or stable disease (SD) lasting for 24 weeks. The secondary endpoints included objective response rate (ORR, defined as the patients with confirmed PR or CR), PFS (defined as the time from enrollment to first documented disease progression or death from any cause), and AEs.

### Statistical analyses

A Simon two-stage optimal design was adopted for this study. The null hypothesis of CBR was 45% [12, 15], and the alternative hypothesis was 64%. With a one-side α of 0.05 and a power of 80%, 17 evaluable patients would be enrolled in the first stage. If more than 8 of 17 patients achieved CBR, the trial would be proceeded to the second stage, and additional 30 evaluable patients would be recruited. If more than 26 among 47 evaluable patients achieved CBR, the treatment would be considered of further interest. Considering 10% of the patients were not evaluable, 53 patients were finally needed.

Efficacy and safety analyses were performed in all patients receiving at least one study medication. The statistical analyses were primarily descriptive. Categorical variables were reported as numbers and percentage, and continuous variables were summarized as median (range). The 95% confidence interval (CI) of CBR and ORR was calculated using the Clopper-Pearson method. PFS was estimated using the Kaplan–Meier method. Statistical analyses were conducted using SAS 9.4.

## Results

### Baseline characteristics of patients

**Fig. 1:**
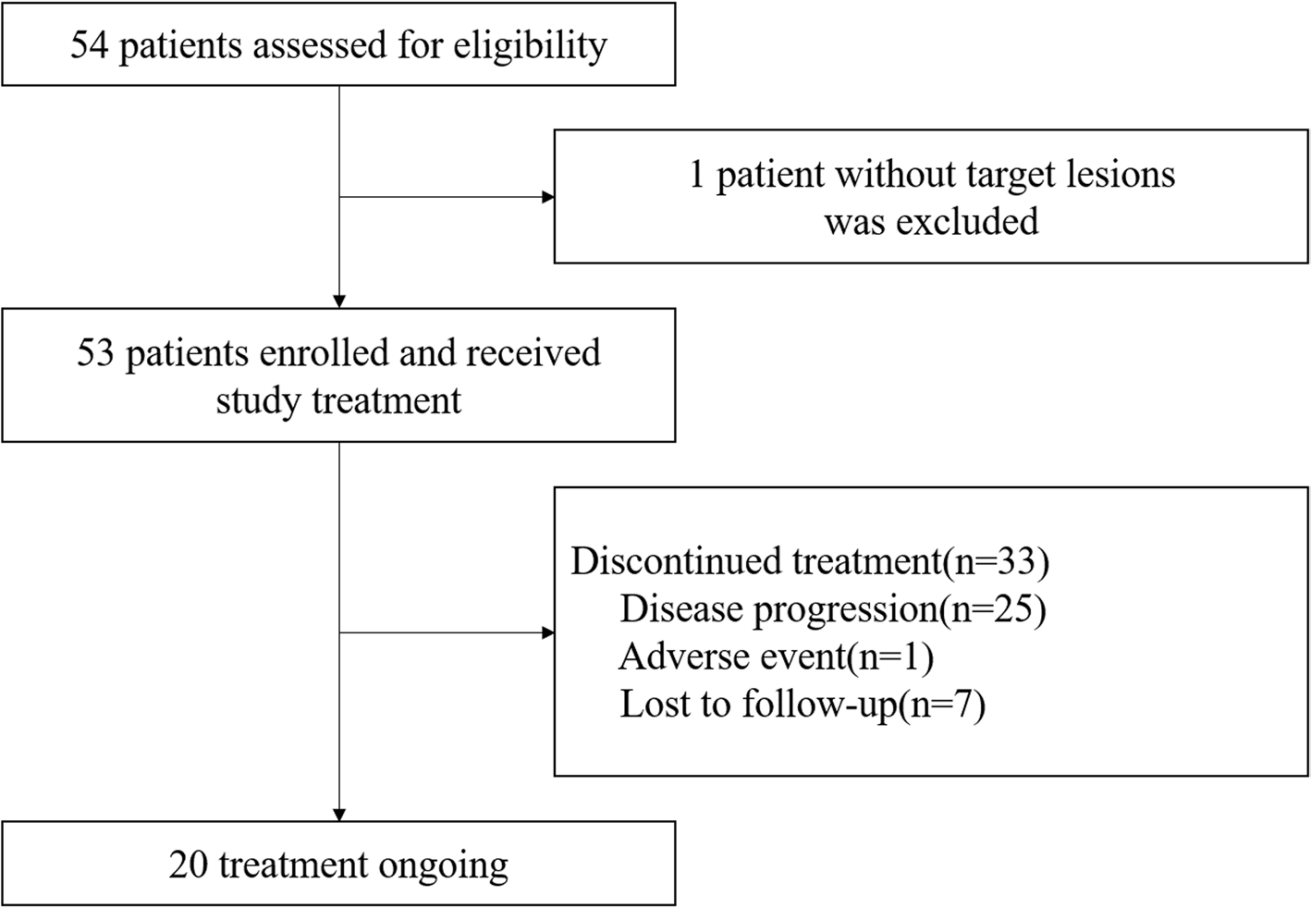
Study flowchart

Between November 2019 and December 2021, 54 patients were assessed for eligibility, and one case without a target lesion was excluded. Finally, 53 patients were enrolled and received study medications (Fig. 1). The median age of all patients was 52 years old (range, 38–69 years old). Twenty-three (43.4%) patients were in premenopausal or perimenopausal period and combined OFS. A total of 18 (34%) patients showed de novo metastases. Sixteen (30.2%) patients developed visceral metastases, and 38 (71.7%) had three or more metastatic sites. Thirty-two (60.4%), 12 (22.6%), and 25 (47.2%) patients had received prior chemotherapy, trastuzumab, and endocrine therapy, respectively (Table 1).

**Table 1:** Baseline characteristics of patients

Characteristics	All (*n* = 53)
Median age, years (range)	52 (38–69)
ECOG performance status, *n* (%)	
0	39 (73.6)
1	14 (26.4)
HER2 expression by IHC, *n* (%)	
2 + and FISH positive	22 (41.5)
3 +	31 (58.5)
Hormone receptor status, *n* (%)	
ER positive and PgR positive	48 (90.6)
ER positive and PgR negative	5 (9.4)
Menopausal status, *n* (%)	
Premenopausal/perimenopausal	23 (43.4)
Postmenopausal	30 (56.6)
Combined with OFS, *n* (%)	
Yes	23 (43.4)
No	30 (56.6)
Disease status, *n* (%)	
De novo disease	18 (34.0)
Recurrent or metastatic disease	35 (66.0)
Number of metastatic sites, *n* (%)	
1	15 (28.3)
2	22 (41.5)
≥ 3	16 (30.2)
Metastatic site, *n* (%)	
Visceral metastasis	38 (71.7)
Non-visceral metastasis	15 (28.3)
Previous (neo)adjuvant therapy, *n* (%)	
Chemotherapy	32 (60.4)
Trastuzumab	12 (22.6)
Endocrine therapy	25 (47.2)
Tamoxifen/toremifene only	20 (37.7)
Aromatase inhibitor only	3 (5.7)
Both	2 (3.8)

*ECOG* Eastern Cooperative Oncology Group, *IHC* immunohistochemistry, *ER* estrogen receptor, *PgR* progesterone receptor, *FISH* fluorescence in situ hybridization, *OFS* ovarian function suppression

### Efficacy

**Fig. 2:**
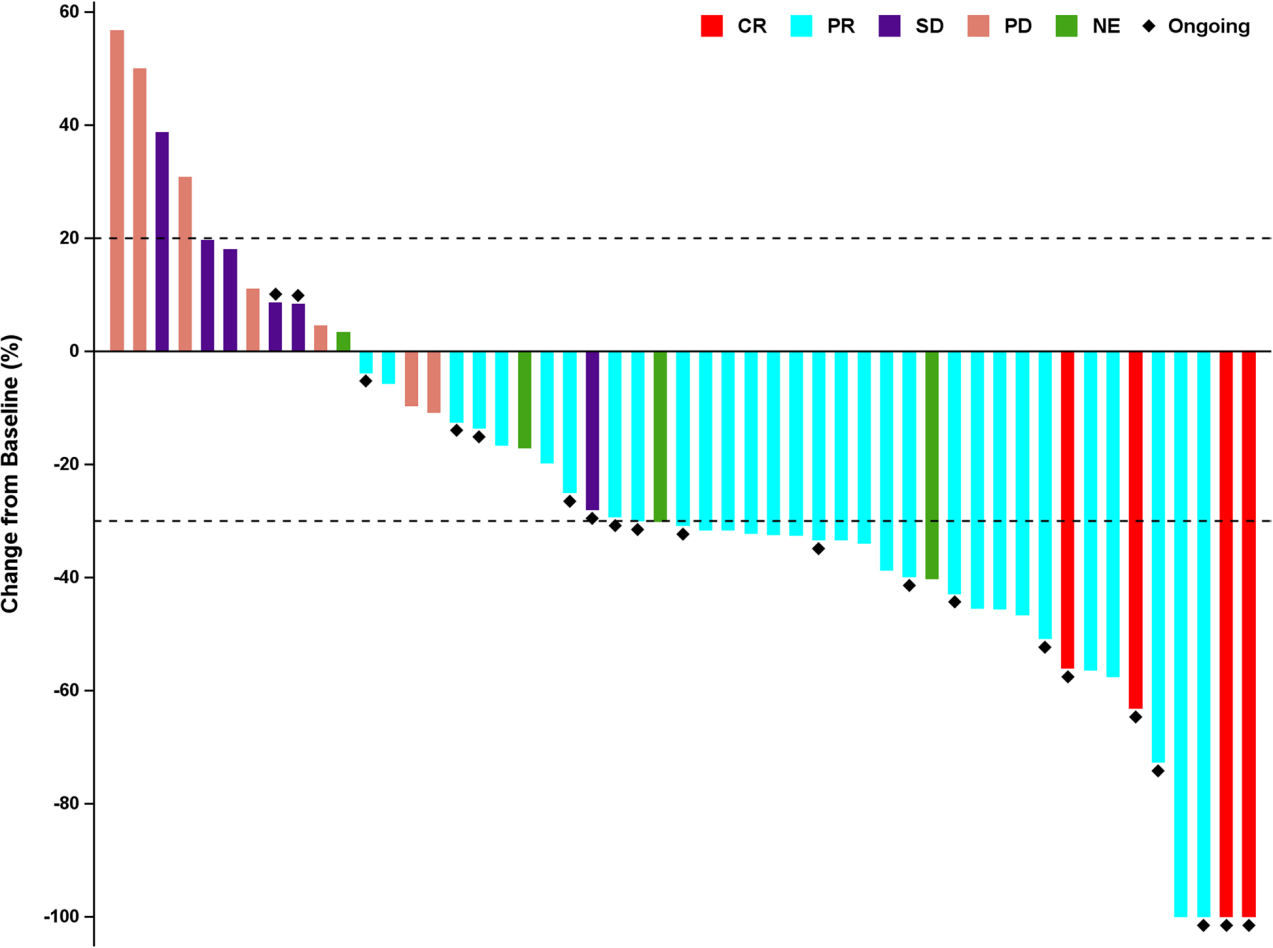
The best percentage changes from baseline in target lesions of patients (*n* = 51). Four patients without confirmation of response were deemed as not evaluable

**Fig. 3:**
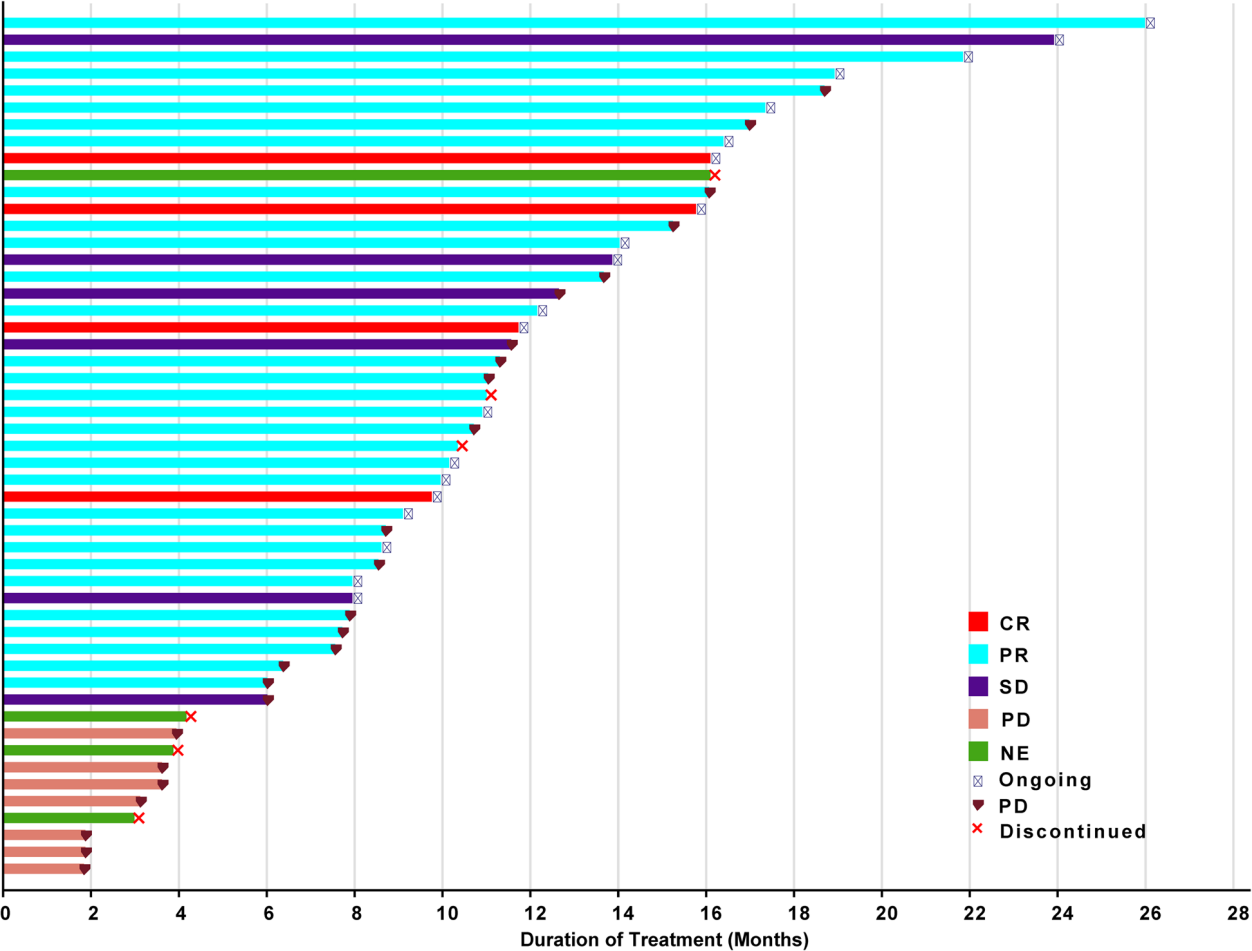
Treatment exposure and response duration of evaluable patients (*n* = 51). Four patients without confirmation of response were deemed as not evaluable

**Fig. 4:**
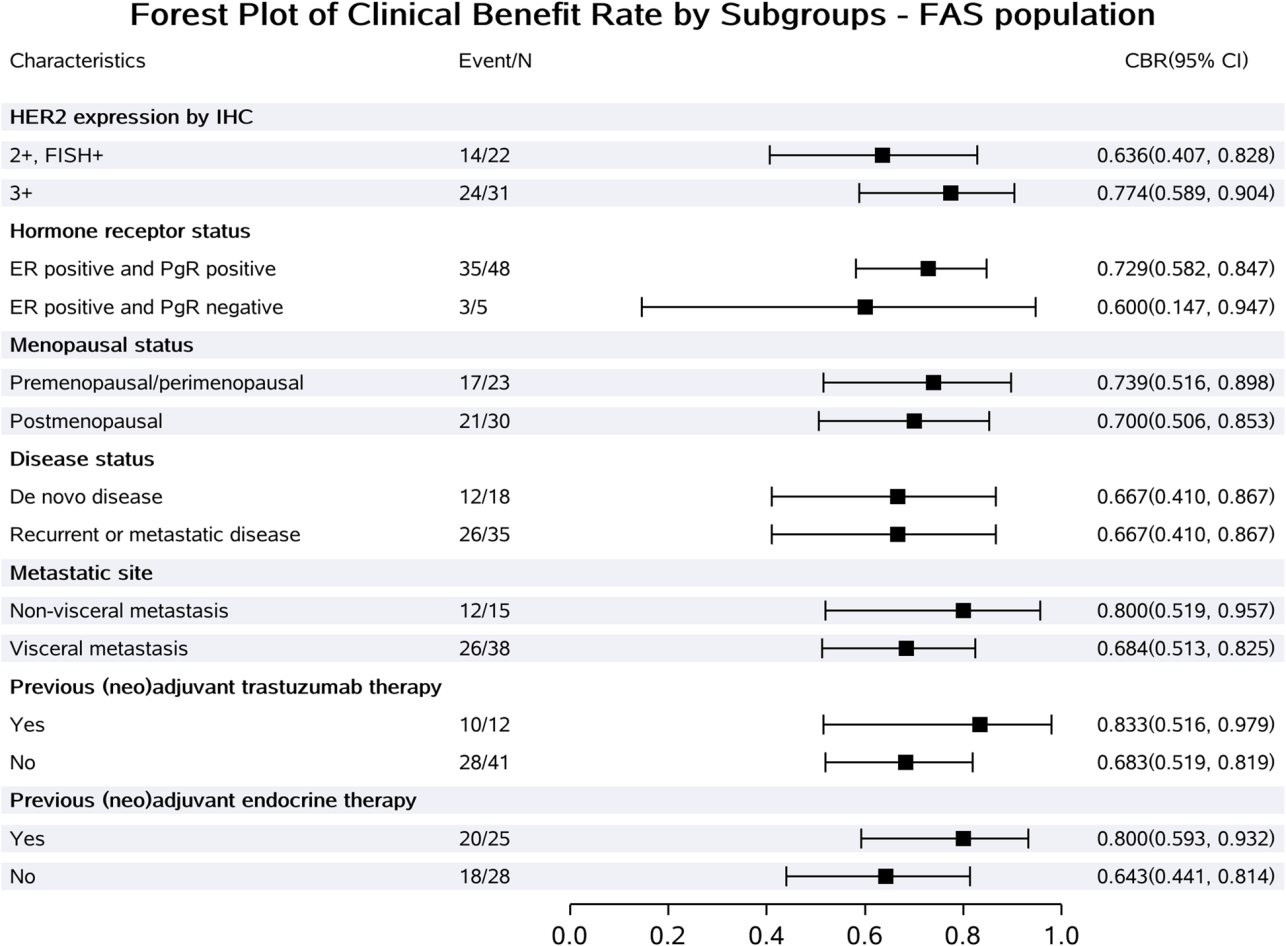
Subgroup analysis of clinical benefit rate

Among all patients, 51 had at least one response evaluation. There were two patients excluded in the first week after the first dose due to protocol violations (not meeting RECIST criteria for response evaluation), and four patients without confirmation of response were deemed as not evaluable. Four (7.5%), 30 (56.6%), and 6 (11.3%) patients achieved confirmed CR, PR, and SD, respectively, giving a CBR of 71.7% (95% CI, 57.7–83.2%), and an ORR of 64.2% (95% CI, 49.8–76.9%) (Table 2, Figs. 2 and 3). The subgroup analysis of CBR is shown in Fig. 4. Generally, the CBR showed consistency across all subgroups.

**Table 2:** Tumor response

Response	All (***n*** = 53)
Best overall response, ***n*** (%)	
Complete response	4 (7.5)
Partial response	30 (56.6)
Stable disease	6 (11.3)
Progressive disease	7 (13.2)
Not evaluable	6 (11.3)
Clinical benefit rate (95% CI)	71.7% (57.7%, 83.2%)
Objective response rate (95% CI)	64.2% (49.8%, 76.9%)

*CI* confidence interval

**Fig. 5:**
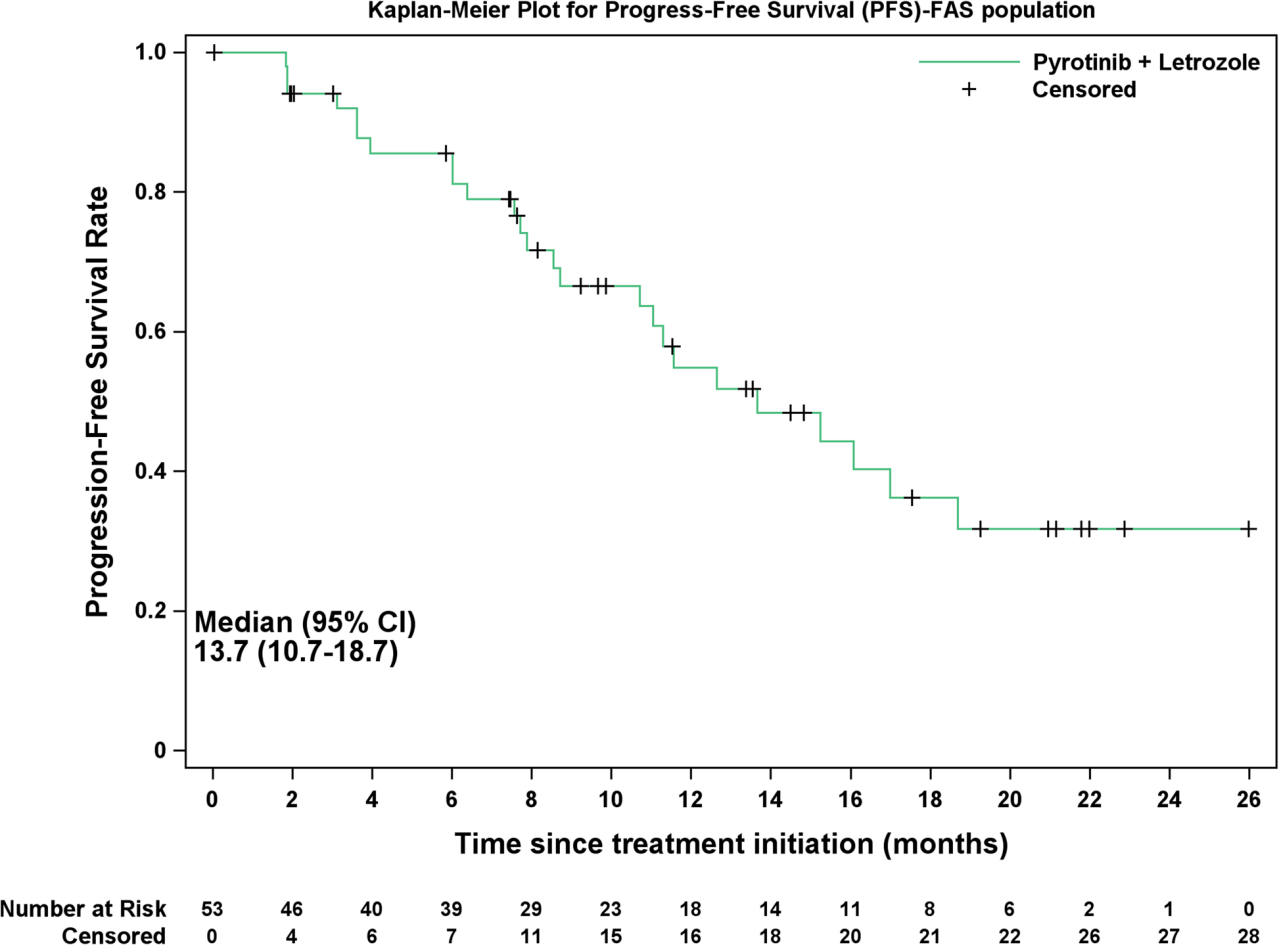
Kaplan–Meier curve of progression-free survival

As of August 2022, the median follow-up duration was 11.6 months (95% CI, 8.7–14.0 months). 33 patients discontinued treatment (25 developed disease progression, one was due to AE and seven lost to follow-up), and treatment was ongoing for 20 patients (Fig. 1). The median PFS was 13.7 months (95% CI, 10.7–18.7 months), and 1-year PFS rate was 54.86% (Fig. 5).

### Safety

No AEs leading to death were reported in our study, and one patient experienced treatment discontinuation due to AEs. The most common treatment-related AEs of any grade were diarrhea (94.3%), hypertriglyceridemia (35.8%), and blood creatinine increased (34.0%). Ten patients (18.9%) developed grade 3 diarrhea (Table 3). No new or unexpected AE was identified, and all AEs were manageable.

**Table 3:** Treatment-related adverse events occurring in at least 5% patients

Events, ***n*** (%)	Any grade	≥ Grade 3
Diarrhea	50 (94.3)	10 (18.9)
Hypertriglyceridemia	19 (35.8)	0
Blood creatinine increased	18 (34.0)	0
Aspartate aminotransferase increased	17 (32.1)	0
Vomiting	16 (30.2)	1 (1.9)
Hyperuricemia	13 (24.5)	0
Alanine aminotransferase increased	12 (22.6)	0
Nausea	11 (20.8)	0
White blood cell count decreased	10 (18.9)	0
Hypercholesterolemia	10 (18.9)	0
Oral ulcer	9 (17.0)	0
Headache	7 (13.2)	0
Urea nitrogen increased	6 (11.3)	0
Anemia	6 (11.3)	0
Hypokalemia	5 (9.4)	0
Rash	5 (9.4)	0
Hand-foot syndrome	5 (9.4)	1 (1.9)
Neutrophil count decreased	5 (9.4)	0
Abdominal distension	4 (7.5)	0
Blood glucose increased	4 (7.5)	0
Paronychia	3 (5.7)	0
Dizziness	3 (5.7)	0
Stomachache	3 (5.7)	0
Appetite decreased	3 (5.7)	0

## Discussion

The current standard first-line treatment for patients with HER2-positive MBC is HER2-targeted therapy plus chemotherapy, regardless of the HR status [3]. However, some patients may not be able to tolerate chemotherapy, and previous studies have also shown the use of chemotherapy is associated with a higher incidence of toxicities than endocrine therapy in HR-positive patients [7, 8]. Besides, some patients cannot seek medical attention in a timely manner, and the oral regimens could improve patients’ compliance. In this phase II study, we investigated a chemo-free oral treatment for these patients. The results showed pyrotinib plus letrozole as the first-line treatment yielded a CBR of 71.7%, an ORR of 64.2%, and a median PFS of 13.7 months. No new safety signal was identified, and all AEs were manageable.

The HER2 and ER signaling pathways cross-talk extensively. As a result of ER signaling, HER2 blockade downstream signaling may be bypassed to facilitate tumor progression [20], resulting in resistance to the therapy [9–11]. Thus, to prevent endocrine resistance, targeted agents have been investigated in combination with endocrine therapy for MBC patients with both HR-positivity and HER2-positivity. TAnDEM study is the first phase III trial without chemotherapy, in which anastrozole and trastuzumab were used for the first-line treatment of postmenopausal HR-positive and HER2-positive MBC [12]. PERTAIN study suggested that the combination of pertuzumab, trastuzumab, and an AI as the first-line treatment improved the prognosis of HR-positive and HER2-positive MBC patients, with a median PFS of 18.89 months (95% CI, 14.09–27.66) [13]. The ALTERNATIVE study showed that trastuzumab plus lapatinib with AI could significantly improve the median PFS, compared to trastuzumab with AI or lapatinib with AI (median PFS: 11.0 months vs. 5.7 and 8.3 months) [21].

Anti-HER2 TKIs also play an important role in HER2-positive breast cancer, and multiple drugs have been approved [14]. However, the use of anti-HER2 TKIs as a first-line treatment for HER2-positive MBC is still a topic of controversy. In the NEfERT-T trial, the median PFS of trastuzumab plus paclitaxel and neratinib plus paclitaxel did not show a significant difference in the first-line treatment of HER2-positive MBC patients (both 12.9 months) [22]. Besides, lapatinib plus taxanes exhibited shorter PFS (9.0 months) and more toxicities compared to trastuzumab plus taxanes for HER2-positive MBC [23]. According to the PANDORA study, the combination of pyrotinib and docetaxel showed benefit in patients with HER2-positive MBC in the first-line setting, with a PFS of 16.0 months [24]. In addition to anti-HER2 TKIs plus chemotherapy, TKIs plus endocrine therapy has also been explored in the HR-positive and HER2-positive MBC. In a study on the treatment of locally recurrent or metastatic HR-positive and HER2-positive MBC with lapatinib and letrozole [15], the CBR was 48%, the ORR was 28%, and the median PFS was 8.2 months. In our study, first-line treatment with pyrotinib and letrozole yielded a CBR of 71.7%, an ORR of 64.2%, and a median PFS of 13.7 months, which was numerically better than that of lapatinib and letrozole. A number of factors contributed to this, including improved HER2 testing technology and increased experience with managing toxicities of targeted therapies among health care professionals [25]. Besides, only Chinese patients were included in this study, which may have different outcomes than other populations [26]. Despite this, previous studies also demonstrated the superiority of pyrotinib over lapatinib when combined with capecitabine in previously treated MBC with HER2-positive disease, which may be due to that pyrotinib is an irreversible pan-HER TKI [17, 18].

In this study, 22% of patients had previously received trastuzumab, and the percentage is 11% in the CLEOPATRA study [4–6], 11% in the PUFFIN study [27], and 15% in the PHILA study [28]. The ALTERNATIVE study required all patients to have received prior treatment with trastuzumab plus chemotherapy, which may explain the lower PFS observed in that study [21]. Interestingly, patients who had previously received trastuzumab had a numerically higher CBR in our study, which was consistent with the findings of the PHILA and PANDORA study [24, 28]. The PHILA study reported that patients who had previously received trastuzumab had a numerically higher PFS than those who had not received prior trastuzumab. However, this finding needs to be further confirmed in future studies due to the small sample size in our study.

In our study, no new or unexpected AE was identified, and all AEs were manageable. Previous studies also suggested that patients with MBC are more likely to experience toxicities after receiving chemotherapy than after receiving endocrine therapy [7, 8]. Consistent with previous studies of pyrotinib, the most common treatment-related AEs was diarrhea in this study [17, 22, 23, 29]. A total of ten patients (18.9%) developed grade 3 diarrhea, which is lower than in previous studies, suggesting that pyrotinib plus AI may be less toxic than pyrotinib plus chemotherapy. Besides, many cancer patients have not been able to receive intravenous drug therapy in time. It is noteworthy that both pyrotinib and letrozole are oral regimens. Patients can administer the treatment at home, reducing the need for hospitalization.

This study has some limitations. First, since this is a single-arm study with a small sample size and no control group, the results might be biased. Second, the OS data is immature now, and long-term follow-up is ongoing. Third, the current standard treatment is a dual-HER2 blockade, and our results can only provide preliminary evidence of pyrotinib in the first-line treatment of HR-positive and HER2-positive MBC patients. Of note, a phase III randomized trial on the pyrotinib, trastuzumab, and an AI for HR-positive and HER2-positive MBC patients is ongoing [30]. Forth, several studies have demonstrated the benefit of CDK4/6 inhibitors in the treatment of HR-positive and HER2-positive patients [31, 32]. However, the role of CDK4/6 inhibitors plus pyrotinib was not investigated in our study. Of note, phase Ib trial LORDSHIPS tested the activity of dalpiciclib combined with pyrotinib and letrozole in HR-positive and HER2-positive MBCs, and the phase II trial is ongoing [33]. Finally, the common AE to HER2 TKIs is diarrhea, and in this study, anti-diarrheal drugs were not administered as prophylaxis. Future studies may investigate the benefit of anti-diarrheal drugs as a primary prophylaxis in similar regimens.

## Conclusion

In conclusion, our preliminary results suggested that pyrotinib plus letrozole is feasible for the first-line treatment of patients with HR-positive and HER2-positive MBC, with a median PFS of 13.7 months. The toxicities were manageable. Subsequent large-scale trials are required to assess the efficacy of pyrotinib plus AI and other anti-HER2 agents or CDK4/6 inhibitors for HR-positive and HER2-positive MBC.

## Data Availability

All datasets generated or analyzed during this trial that supporting the results are available from the corresponding author upon reasonable request.

## Abbreviations

AEs: Adverse events
AI: Aromatase inhibitor
CBR: Clinical benefit rate
CI: Confidence interval
CNS: Central nervous system
CR: Complete response
ECOG: Eastern Cooperative Oncology Group
ER: Estrogen receptor
HER2: Human epidermal growth factor receptor 2
HR: Hormone receptor
MBC: Metastatic breast cancer
NCI-CTCAE: The National Cancer Institute Common Terminology Criteria for Adverse Events
OFS: Ovarian function suppression
ORR: Objective response rate
OS: Overall survival
PFS: Progression-free survival
PR: Partial response
RECIST: The Response Evaluation Criteria in Solid Tumors
SD: Stable disease
TKI: Tyrosine kinase inhibitor
